# Reprogramming the immunosuppressive microenvironment in glioblastoma: the microglia–MDSC–Treg axis and translational therapeutic strategies

**DOI:** 10.3389/fonc.2026.1782775

**Published:** 2026-08-10

**Authors:** Hasan Ali Aydın, Emrah Keskin, Murat Kalaycı

**Affiliations:** Department of Neurosurgery, Faculty of Medicine, Zonguldak Bülent Ecevit University, Zonguldak, Türkiye

**Keywords:** cancer immunotherapy, glioblastoma, immunometabolism, microglia, myeloid immunity, myeloid-derived suppressor cells, regulatory T cells, tumor microenvironment

## Abstract

Glioblastoma (GBM) remains one of the most lethal primary brain tumors despite maximal surgical resection, radiotherapy, and temozolomide. Immune checkpoint inhibitors have failed to demonstrate durable benefit in three large phase III trials (CheckMate 143, 498, and 548), underscoring profound, multilayered immune resistance. This narrative review synthesizes the immunosuppressive GBM microenvironment, situating the microglia–myeloid-derived suppressor cell (MDSC)–regulatory T cell (Treg) axis within the broader immune landscape, including dendritic cells, natural killer cells, exhausted CD8^+^ T cells, neutrophils, and B cells. We distinguish ontogenetically distinct resident microglia from bone marrow–derived macrophages, separate monocytic (M-MDSC) from polymorphonuclear (PMN-MDSC) subsets, and examine how radiotherapy reshapes immunity. We extend the immunometabolic discussion beyond indoleamine 2,3-dioxygenase (IDO) to the adenosine (CD39/CD73/A2A), arginine, hypoxia, lactate, and glutamine pathways, and critically analyze why checkpoint blockade has failed. We summarize emerging strategies, including CSF1R, CCR2, CXCR2, CD47–SIRPα, STING, CD40, TGF-β, and IL-1β targeting, together with candidate biomarkers such as circulating MDSCs, CSF1, IL-1β, multiplex immunofluorescence, spatial transcriptomics, and single-cell RNA sequencing, for rational patient selection. We frame these mechanisms as a single, self-reinforcing circuit integrating myeloid-driven immune regulation, metabolic reprogramming, and treatment-induced immune remodeling. We argue that future progress depends on biomarker-driven combinations that reprogram the myeloid compartment, relieve metabolic suppression, and actively inflame the tumor, integrated with radiotherapy and tailored to molecular context (IDH and MGMT status).

## Introduction

Glioblastoma represents the most aggressive primary malignancy of the central nervous system, with a median survival of approximately 15 months despite aggressive multimodal therapy (1, 2). While immunotherapy has transformed outcomes in several solid tumors, GBM has remained largely refractory (3, 4). Early hypotheses attributed this resistance to the blood–brain barrier and immune privilege; however, mounting evidence indicates that GBM actively establishes a deeply immunosuppressive tumor microenvironment (TME) (4, 5).

Unlike immunogenic tumors driven predominantly by dysfunctional T-cell responses, GBM is characterized by a myeloid-dominant immune landscape in which microglia, macrophages, and MDSCs outnumber lymphocytes and orchestrate immune evasion (6, 7). These cells interact dynamically with regulatory T cells to suppress antigen presentation, metabolically incapacitate effector T cells, and promote immune tolerance (8). Importantly, this immune landscape does not exist in isolation from tumor genetics: isocitrate dehydrogenase (IDH) mutation status, O^6^-methylguanine-DNA methyltransferase (MGMT) promoter methylation, and histological grade are established prognostic determinants that also shape the composition and density of the immune infiltrate, contributing to the intratumoral heterogeneity that limits translation (9, 10). IDH-mutant gliomas, for example, harbor a comparatively less inflamed, less myeloid-rich microenvironment than IDH-wild-type GBM, with direct implications for immunotherapy responsiveness (11, 12).

Understanding and therapeutically targeting this integrated network is essential for advancing immunotherapy beyond the current standard of care (13). We propose that immune suppression in glioblastoma is best understood not as a set of independent defects but as a single, self-reinforcing circuit organized around three interconnected axes: myeloid-driven immune regulation, encompassing microglia, macrophages, and MDSCs; metabolic reprogramming of the microenvironment, spanning the tryptophan, adenosine, arginine, hypoxia, lactate, and glutamine pathways; and treatment-induced immune remodeling, principally the local and systemic effects of radiotherapy. These axes are mutually amplifying, operate on a background set by IDH and MGMT status, and together explain why interventions directed at a single node have failed.

**Figure 1:**
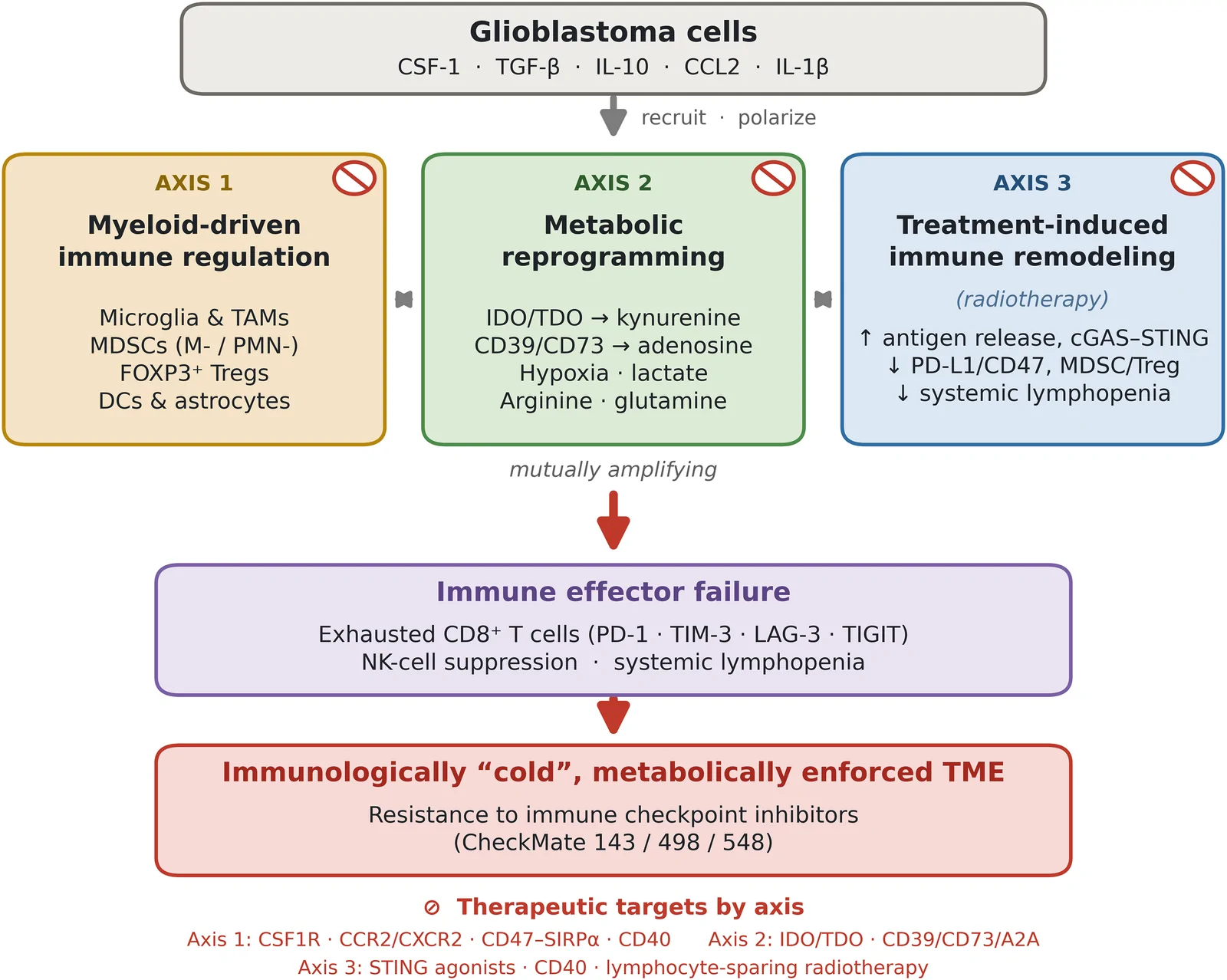
The three-axis model of immune suppression in glioblastoma. Glioblastoma cells secrete CSF-1, TGF-β, IL-10, CCL2, and IL-1β, which recruit and polarize the suppressive microenvironment. Three mutually amplifying axes sustain immune suppression. Axis 1, myeloid-driven immune regulation, comprises microglia and bone marrow–derived macrophages (TAMs) in an M0-like, poorly antigen-presenting state; monocytic (M-MDSC, CD14^+^) and polymorphonuclear (PMN-MDSC, CD15^+^) myeloid-derived suppressor cells acting through arginase-1, nitric oxide, and reactive oxygen species; FOXP3^+^ regulatory T cells; and dysfunctional dendritic cells together with glioblastoma-instructed reactive astrocytes. Axis 2, metabolic reprogramming, comprises the IDO/TDO tryptophan–kynurenine pathway, the CD39/CD73/A2A adenosine cascade, and hypoxia, lactate, arginine, and glutamine metabolism. Axis 3, treatment-induced immune remodeling, reflects the dual effect of radiotherapy, which releases tumor antigen and activates cGAS–STING with type I interferon signaling while upregulating PD-L1 and CD47, recruiting MDSCs and Tregs, and producing systemic lymphopenia. Bidirectional arrows indicate that the axes reinforce one another. Their convergence produces immune effector failure, with exhausted CD8^+^ T cells co-expressing PD-1, TIM-3, LAG-3, and TIGIT, suppressed natural killer cells, and systemic lymphopenia, yielding an immunologically “cold”, metabolically enforced microenvironment that underlies resistance to immune checkpoint inhibitors (CheckMate 143, 498, and 548). Candidate therapeutic targets are grouped by axis and marked with red no-entry badges.

The review is organized around this model: after surveying the full immune cellular landscape, we examine each axis in turn, analyze the reasons for checkpoint-inhibitor failure, and derive candidate biomarkers and rational combination strategies from the model itself (**Figure 1**).

## Methods: literature search strategy

This is a narrative review. We searched PubMed/MEDLINE and Web of Science for English-language articles published between January 2000 and June 2026, combining “glioblastoma” or “glioma” with “tumor microenvironment,” “microglia,” “macrophage,” “myeloid-derived suppressor cell,” “regulatory T cell,” “immunometabolism,” “immune checkpoint inhibitor,” “CSF1R,” “adenosine,” “radiotherapy,” “single-cell RNA sequencing,” and “spatial transcriptomics.” Reference lists of key reviews and the ClinicalTrials.gov registry were screened for additional trials. Priority was given to mechanistic studies, randomized controlled trials, and recent single-cell and spatial analyses. Selection was made by the authors on the basis of relevance and scientific quality rather than a formal systematic protocol, consistent with the narrative format; this is acknowledged as a limitation.

## The glioblastoma immune microenvironment: a myeloid-dominant but multicellular landscape

Microglia and infiltrating macrophages collectively constitute a large fraction of the cellular content of glioblastomas. Estimates vary with species and methodology: human studies generally report glioma-associated microglia/macrophages comprising roughly 30–50% of the tumor mass, whereas murine models can show comparable or higher proportions, so the frequently cited “up to 50%” figure represents an upper bound derived predominantly from human histological and flow-cytometric series rather than a fixed value (6, 14). These cells exhibit functional plasticity and are actively polarized toward an immunosuppressive phenotype by glioma-derived cytokines such as CSF-1, IL-10, and TGF-β (11, 15, 16). The principal immunosuppressive populations and their dominant mechanisms are summarized in **Table 1**. Rather than functioning as effective antigen-presenting cells, glioma-associated microglia demonstrate reduced expression of co-stimulatory molecules and impaired T-cell activation capacity (14).

**Table 1:** Key immunosuppressive cell populations in the glioblastoma microenvironment, distinguishing resident microglia from bone marrow–derived macrophages and monocytic (M-MDSC) from polymorphonuclear (PMN-MDSC) subsets.

Cell population	Cellular origin	Dominant immunosuppressive mechanisms	Functional impact on antitumor immunity
Resident microglia	Yolk-sac–derived CNS myeloid cells	Reduced co-stimulatory and MHC-II expression; IL-10 and TGF-β secretion; checkpoint-ligand expression	Impaired antigen presentation and T-cell activation; tolerance
Infiltrating/bone marrow–derived macrophages (TAMs)	Circulating monocytes (bone marrow)	CSF-1–driven pro-tumoral polarization; ECM remodeling; angiogenic and immunosuppressive cytokines	Immune exclusion, invasion, resistance to immunotherapy
M-MDSCs (monocytic)	Peripheral immature myeloid cells	Arginase-1, iNOS/NO (CD14^+^); male-associated; intratumoral enrichment in murine models	Antigen-non-specific suppression of T-cell proliferation
PMN-MDSCs (polymorphonuclear)	Peripheral immature myeloid cells	ROS, arginase-1 (CD15^+^); predominate within human tumor tissue; female-associated	Suppression of effector T cells; systemic dysfunction
Regulatory T cells (Tregs)	CD4^+^ T lymphocytes	CTLA-4; IL-10 and TGF-β; IL-2 consumption; adenosine (CD39/CD73)	Direct suppression of cytotoxic T-cell responses

### Resident microglia versus bone marrow–derived macrophages

A recurring source of imprecision in the GBM literature is the conflation of resident microglia with infiltrating macrophages. These populations are ontogenetically distinct: microglia arise from yolk-sac progenitors and self-renew within the CNS, whereas tumor-associated macrophages (TAMs) derive from circulating bone marrow monocytes recruited across a disrupted blood–brain barrier (15). Lineage-tracing and single-cell studies show that they occupy different spatial niches and adopt different transcriptional programs, with monocyte-derived macrophages enriched perivascularly and in necrotic/hypoxic regions and generally displaying a more immunosuppressive, pro-angiogenic profile, while microglia predominate at the infiltrative margin (6, 15). Many glioma-associated myeloid cells co-express pro- and anti-inflammatory markers, resembling an M0-like state rather than a clean M1/M2 dichotomy (17). This distinction is therapeutically consequential, because agents that deplete or reprogram one compartment may spare, or paradoxically expand, the other; throughout this review we therefore specify “microglia” and “infiltrating/bone marrow–derived macrophages,” reserving “TAMs” for the mixed compartment.

### Myeloid-derived suppressor cells: monocytic and polymorphonuclear subsets

MDSCs are a heterogeneous population of immature myeloid cells that are elevated in the peripheral blood of GBM patients relative to those with low-grade CNS tumors and accumulate within the tumor, where greater infiltration associates with worse outcome (18, 19). Two principal subsets differ in biology, suppressive mechanism, and therapeutic vulnerability: monocytic MDSCs (M-MDSCs; CD14^+^ in humans), which suppress predominantly through arginase-1 and inducible nitric oxide synthase–derived nitric oxide, and polymorphonuclear/granulocytic MDSCs (PMN-MDSCs; CD15^+^), which act largely via reactive oxygen species and arginase-1. In human GBM both subsets are increased in the circulation, whereas PMN-MDSCs predominate within the tumor tissue; notably, the opposite pattern (intratumoral M-MDSC enrichment) is observed in murine models, so the dominant intratumoral subset remains species- and context-dependent (20, 21). Reported prevalence varies with markers and methodology, but circulating MDSCs are typically increased several-fold over controls and constitute a measurable, prognostically relevant fraction of the myeloid infiltrate.

This compartmentalization has direct implications for marker selection and for the choice of depleting agent (for example, CXCR2 blockade for granulocytic cells versus CCR2/CSF1R-directed approaches for the monocytic lineage). Sex-specific differences further refine this picture: monocytic MDSCs are enriched within tumors of male patients, whereas granulocytic MDSCs are more prominent and prognostically adverse in females, paralleling the recognized prognostic interaction between patient sex and MGMT status and suggesting that MDSC-directed therapy may need to be tailored by subset and by sex (10, 21). Through these mechanisms MDSCs suppress T-cell proliferation and function and reinforce immune suppression indirectly by promoting Treg expansion and stabilizing suppressive macrophage phenotypes, positioning them as central amplifiers of immune dysfunction (19).

### Regulatory T cells

Regulatory T cells are consistently enriched within glioblastoma tissue and, across most series, correlate with poor clinical outcomes, although associations in GBM are less uniform than in some extracranial tumors (22). Their suppressive activity is enhanced by myeloid-derived cytokines and by metabolic constraints within the TME, including adenosine generated through the CD39/CD73 ectonucleotidase cascade expressed by both Tregs and myeloid cells (23). The interaction between Tregs and myeloid cells forms a self-reinforcing loop that limits adaptive immunity even in the presence of tumor antigens, and the intratumoral CD8^+^/Treg ratio may carry stronger prognostic information than Treg density alone.

### Dendritic cells, NK cells, CD8^+^ T cells, neutrophils, B cells, and reactive astrocytes

Although the GBM microenvironment is myeloid-dominant, several additional populations shape the balance between suppression and response (7). Dendritic cells are scarce and frequently dysfunctional in GBM, with impaired maturation and antigen presentation that weakens priming of tumor-specific T cells and provides one rationale for dendritic-cell vaccines and CD40 agonists. Natural killer cells can lyse glioma stem cells but are restrained by TGF-β, adenosine, and inhibitory receptor–ligand signaling. CD8^+^ cytotoxic T cells, when present, frequently display an exhausted phenotype with co-expression of PD-1, TIM-3, LAG-3, and TIGIT, and are further depleted by treatment-related lymphopenia and bone-marrow sequestration of naïve T cells (8).

Tumor-associated neutrophils, overlapping phenotypically with PMN-MDSCs, contribute pro-angiogenic and immunosuppressive functions and have been linked to anti-angiogenic resistance and worse outcomes. B cells and regulatory B cells are comparatively understudied but can contribute through IL-10 and antibody-mediated mechanisms. Beyond classical immune cells, glioblastoma-instructed reactive astrocytes have recently been shown to actively suppress tumor-specific T-cell immunity, adding a non-myeloid stromal layer to the suppressive network (24). Spatially resolved analyses confirm that these populations are not randomly distributed: hypoxia organizes the tissue into reproducible niches in which an inflammatory, MHC-class-II–low macrophage program colocalizes with exhausted CD8^+^ T cells (25), and immunophenotypic profiling of IDH-wild-type GBM cohorts has begun to define the dominant suppressive cell states (12).

### CSF1R signaling and adaptive resistance

CSF1R signaling is central to the survival and polarization of microglia and TAMs (16). Preclinical CSF1R inhibition reprogrammed macrophages toward a less suppressive phenotype and prolonged survival in glioma models (16), yet monotherapy showed limited clinical efficacy (26). The mechanism of acquired resistance has since been defined: surviving macrophages are re-educated by tumor- and stroma-derived signals (including IGF-1 and PI3K activation), restoring a protumoral state despite continued blockade (27); combination with PD-1 blockade has been explored to counter this (28). These findings reframed CSF1R inhibition from a standalone strategy to a combination partner.

## Radiotherapy and the immune microenvironment

The suppressive network described above does not operate in a therapeutic vacuum, and the treatment every patient receives continuously reshapes it, which brings the third axis of the model into view. Radiotherapy is a standard component of GBM treatment and exerts profound, bidirectional effects on tumor immunity that are essential to any translational discussion, particularly because most immunotherapy trials combine the two. Ionizing radiation induces immunogenic cell death, releases tumor-associated antigens and damage-associated molecular patterns, activates cytosolic DNA sensing through the cGAS–STING pathway with type I interferon production, and can enhance MHC-I expression and antigen presentation, effects that underpin the rationale for radio-immunotherapy (29, 30).

Conversely, radiotherapy reinforces immunosuppression: it promotes recruitment and M2-like polarization of macrophages and MDSCs through CCL2/CCL8 and related chemokine signaling, expands intratumoral Tregs, and upregulates immune-checkpoint and “don’t-eat-me” ligands including PD-L1 and CD47 on tumor and myeloid cells (30). Critically, conventionally fractionated cranial radiotherapy, often compounded by corticosteroids and temozolomide, produces severe, durable systemic lymphopenia that depletes the very effector cells immunotherapy seeks to mobilize (4). The net immunological effect is therefore dose-, fraction-, and timing-dependent, which explains why simply adding a checkpoint inhibitor to chemoradiation has underperformed and motivates strategies that exploit radiotherapy-elicited antigen release and STING activation while blocking the compensatory CD47/PD-L1 and adenosinergic programs it induces (30).

Beyond effects within the irradiated field, radiotherapy has systemic immunological consequences that are increasingly recognized as barriers to immunotherapy. Severe treatment-related lymphopenia is common after chemoradiotherapy and is independently associated with worse survival in GBM, as confirmed in a pooled analysis of more than 1,700 patients (43). This lymphopenia does not arise solely from direct cytotoxicity to circulating lymphocytes; cranial irradiation also drives reactive myelopoiesis in the bone marrow, expanding circulating MDSCs whose regulatory gene programs track with the depth of lymphopenia, and depleting these cells in preclinical models abrogates lymphopenia and improves survival (42).

Radiation-induced cytokine signaling further skews hematopoiesis toward suppressive myeloid output, while radio-resistant M-MDSCs upregulate PD-L1, reinforcing suppression at the very moment effector lymphocytes are being lost (30, 42). Conventionally fractionated radiotherapy can therefore blunt the systemic immune competence on which checkpoint blockade depends, which argues for lymphocyte-sparing planning, careful attention to the timing of immunotherapy relative to radiation, and myeloid-directed strategies that decouple the antigenic benefits of radiotherapy from its systemic immunosuppressive cost (42, 43).

## Immunometabolic checkpoints

If the myeloid compartment provides the cellular machinery of immune suppression and radiotherapy modulates it, the second axis supplies its biochemistry. Metabolic reprogramming of the GBM microenvironment is a major, targetable axis of immune suppression that extends well beyond the tryptophan pathway.

### Tryptophan–kynurenine (IDO/TDO) axis

IDO-mediated tryptophan catabolism depletes tryptophan and generates kynurenine, inducing T-cell anergy and promoting Treg differentiation via the aryl hydrocarbon receptor (31, 32). IDO inhibitors have nonetheless produced disappointing clinical results. A key mechanistic reason is redundancy within the pathway: tryptophan-2,3-dioxygenase (TDO2) is independently expressed by gliomas and can sustain kynurenine production when IDO1 is inhibited, so selective IDO1 blockade may be insufficient and dual IDO/TDO or downstream AhR-directed approaches are being explored (33).

### Adenosine (CD39/CD73/A2A) pathway

Hypoxia and extracellular ATP drive the CD39/CD73 ectonucleotidase cascade, generating adenosine that signals through A2A/A2B receptors to suppress effector T-cell and NK-cell function, blunt dendritic-cell maturation, and promote M2 macrophage and Treg phenotypes. Spatially resolved analyses show that hypoxic niches organize this immunosuppressive program in glioma, with monocyte-derived macrophages concentrated in hypoxic regions where adenosinergic and checkpoint programs converge (25). The adenosine axis is therefore an attractive combination target, particularly alongside radiotherapy and checkpoint blockade.

### Arginine, hypoxia, lactate, and glutamine

Arginine metabolism by arginase-1–expressing MDSCs and TAMs depletes a substrate essential for T-cell-receptor signaling and proliferation; reprogramming myeloid arginine metabolism from an arginase-1^+^ to an inducible nitric oxide synthase^+^ state has been shown to relieve this metabolic brake and restore effector T-cell function in brain-tumor models (30). Pervasive hypoxia stabilizes HIF-1α, amplifying VEGF, CCL2, adenosine, and lactate production and acting as a dominant spatial organizer of the suppressive microenvironment (25). Tumor-derived lactate acidifies the microenvironment, polarizes macrophages toward an M2 phenotype, stabilizes FoxP3 in Tregs, and further enhances CD39/CD73 expression, linking the glycolytic and adenosinergic programs, while glutamine addiction of glioma cells competes with infiltrating lymphocytes for a critical nutrient. Together these pathways form an interlocking metabolic barrier that complements the cellular and cytokine-mediated mechanisms above and argues for metabolic–immune combination therapy.

## Immune-activating platforms: oncolytic viral therapies

Oncolytic viruses (OVs) directly lyse tumor cells while activating innate and adaptive immunity (29). Clinical trials using poliovirus, adenovirus, and herpes simplex virus–based platforms have demonstrated safety and immune activation in GBM patients, with occasional durable responders (34, 35). OVs can partially overcome myeloid-driven suppression by enhancing antigen release and inflammatory cytokine production, but corticosteroid use, limited intratumoral spread, and antiviral immunity constrain efficacy, positioning OVs as immune-priming platforms best deployed within rational combinations rather than as standalone therapies (36).

## Why have immune checkpoint inhibitors failed in glioblastoma?

With the three axes now described, their combined clinical consequence can be examined directly, because checkpoint blockade is the intervention that has tested the microenvironment most severely. The clinical record of checkpoint blockade in GBM is now defined by three large, negative phase III trials, and understanding why they failed is central to designing better combinations. CheckMate 143 compared nivolumab with bevacizumab in recurrent GBM and showed no overall-survival benefit (median OS 9.8 vs 10.0 months) (3). CheckMate 498 added nivolumab to radiotherapy in newly diagnosed MGMT-unmethylated GBM and not only missed its primary endpoint but showed inferior survival to temozolomide plus radiotherapy (median OS 13.4 vs 14.9 months), indicating that nivolumab is not a substitute for temozolomide (37). CheckMate 548 added nivolumab to standard chemoradiation in MGMT-methylated GBM and likewise failed to prolong progression-free or overall survival (38).

Several converging mechanisms explain these results. GBM is an immunologically “cold” tumor with relatively low tumor mutational burden and few neoantigens, which limits the targetable T-cell specificities available to checkpoint blockade (5). Even where T cells are present, the myeloid-dominant, metabolically suppressive microenvironment renders them functionally exhausted or excluded, so releasing the PD-1 brake achieves little when the accelerator is also disabled (8). Compounding this, treatment-related and corticosteroid-driven lymphopenia depletes effector T cells; dexamethasone, widely used for peritumoral edema, is profoundly immunosuppressive, and subgroup analyses of corticosteroid-free patients did not rescue the result (37, 38). A further obstacle is the marked intratumoral and interpatient heterogeneity, layered on IDH and MGMT status, which means that unselected enrollment dilutes any responder signal (9, 12). Together, these factors argue that checkpoint blockade cannot succeed as monotherapy and must be embedded in combinations that reprogram myeloid cells, relieve metabolic suppression, and inflame the tumor, with molecularly informed patient selection (4, 13).

## Emerging therapeutic strategies

If a single brake cannot be released in isolation, the therapeutic question becomes which brakes to release together, and the three-axis model provides the frame for that question. The translational strategies targeting the immunosuppressive network, with intended effects, key limitations, and stage of development, are summarized in **Table 2**. Beyond CSF1R inhibition, several myeloid- and metabolism-directed approaches are under investigation. Chemokine-axis blockade (CCL2–CCR2, dual CCR2/CCR5, and CXCR2) aims to interrupt recruitment of monocytic and granulocytic MDSCs and Tregs, though chemokine redundancy has limited single-agent activity (23). The CD47–SIRPα “don’t-eat-me” checkpoint can be blocked to restore macrophage phagocytosis and antigen cross-presentation; anti-CD47 strategies show antitumor activity against glioma and glioma stem cells in preclinical models (39). STING agonists induce type I interferon and repolarize myeloid cells toward an effector state, CD40 agonists license antigen-presenting cells, and TGF-β and IL-1β/inflammasome targeting address dominant suppressive cytokine programs. A particularly instructive proof of concept couples several of these mechanisms with radiotherapy: a bispecific lipid nanoparticle bridging tumor CD47/PD-L1 to myeloid cells and delivering a STING agonist reprogrammed tumor-associated myeloid cells (including an arginase-1^+^ to iNOS^+^ metabolic switch), induced T-cell infiltration, and potentiated radiotherapy in murine GBM, turning radiotherapy-elicited CD47/PD-L1 upregulation into a therapeutic opportunity (30). These data reinforce the thesis that combination, context-dependent reprogramming, rather than single-agent immune release, is required.

**Table 2:** Emerging translational therapeutic strategies targeting the immunosuppressive network in glioblastoma, with intended effect, key translational limitation, and stage of development.

Strategy	Primary target	Intended immunological effect	Key limitation in translation	Development stage
CSF1R inhibitors (e.g., pexidartinib, BLZ945)	Microglia/TAMs	Myeloid depletion or reprogramming to a less suppressive state	Adaptive resistance; compensatory signaling; fibrotic remodeling	Phase II (negative as monotherapy); preclinical combinations
CCL2–CCR2/CCR2–CCR5 blockade	Monocyte/MDSC/Treg recruitment	Reduced myeloid and Treg trafficking to the tumor	Chemokine redundancy; limited single-agent activity	Phase I–II
CXCR2 inhibition	PMN-MDSCs/neutrophils	Blockade of granulocytic MDSC recruitment	On-target neutropenia; preclinical evidence in glioma	Preclinical
CD47–SIRPα inhibitors	Macrophage “don’t-eat-me” checkpoint	Restoration of phagocytosis and antigen cross-presentation	On-target anemia; limited CNS data; antigen sink	Preclinical in glioma; Phase I (other solid tumors)
STING agonists	Innate sensing in myeloid cells	Type I IFN; myeloid repolarization; “cold-to-hot” conversion	Delivery across the blood–brain barrier; systemic toxicity	Preclinical/early Phase I
CD40 agonists	Antigen-presenting cells	Licensing of dendritic cells; macrophage activation	Hepatotoxicity; cytokine-related effects	Phase I–II
TGF-β inhibition	Immunosuppressive cytokine axis	Relief of T-cell suppression; reduced invasion	Pleiotropic toxicity; narrow therapeutic window	Phase I–II (galunisertib)
IL-1β/inflammasome targeting	Myeloid inflammatory signaling	Reduced MDSC recruitment and protumoral inflammation	Indirect effect; biomarker selection needed	Preclinical
IDO/TDO pathway inhibitors	Tryptophan–kynurenine axis	Restoration of effector T-cell function; reduced Treg differentiation	TDO2 redundancy; negative as monotherapy	Phase I–II
Adenosine (CD39/CD73/A2A) blockade	Immunometabolic checkpoint	Reversal of metabolic T- and NK-cell exhaustion	Hypoxia-driven redundancy; combination required	Phase I–II (extracranial)
Oncolytic viruses	Tumor cells + innate immunity	Tumor lysis; antigen release; type I IFN; priming	Limited spread; corticosteroid-induced suppression	Phase I–II
Treg- and MDSC-modulating approaches	Suppressive lymphoid/myeloid compartments	Reduced intratumoral suppression	Autoimmunity risk; heterogeneity; lack of selective markers	Preclinical/Phase I

Framing these agents through the three-axis model clarifies how they might be combined rationally rather than tested in isolation. Because each axis reinforces the others, the most promising regimens pair interventions with complementary mechanisms. A myeloid-repolarizing or MDSC-depleting agent (for example CSF1R, CCR2, or arginase inhibition) can lift the dominant myeloid brake, and combining it with checkpoint blockade licenses the effector T cells thereby unmasked, addressing the observation that anti-PD-1 monotherapy fails when the myeloid compartment keeps effector cells excluded or exhausted. Layering a metabolic intervention such as adenosine-axis or IDO/TDO blockade relieves the biochemical suppression that persists after myeloid reprogramming, and an inflammatory or antigenic stimulus (STING or CD40 agonism, an oncolytic virus, or appropriately timed radiotherapy) supplies the priming signal that an antigen-poor tumor otherwise lacks. Radiotherapy is a natural anchor for such combinations because it simultaneously releases antigen and activates cGAS–STING while inducing the compensatory CD47/PD-L1 and myeloid programs that a co-administered myeloid- or checkpoint-directed agent is designed to block (30, 42). The organizing principle is mechanistic complementarity across the three axes, delivered with attention to sequence and timing, rather than the empirical stacking of agents that act on the same node.

## Biomarkers for patient selection and response monitoring

The repeated failure of unselected trials makes biomarker-guided selection a priority, and candidate markers can be grouped into three complementary tiers. Circulating, blood-based biomarkers are the most accessible: MDSCs resolved into M- and PMN-MDSC subsets correlate with tumor burden and may serve as pharmacodynamic readouts for myeloid-directed therapy, and treatment-related lymphopenia together with circulating MDSC programs provides a window onto radiotherapy-driven systemic remodeling (18, 20, 42).

Tissue-based biomarkers add mechanistic and spatial resolution: soluble and tissue mediators such as CSF1, IL-1β, and kynurenine index myeloid and metabolic suppression, while multiplex immunofluorescence and imaging mass cytometry capture not only the abundance but the spatial organization of immune cells, such as the proximity of suppressive myeloid niches to exhausted CD8^+^ T cells, which carries prognostic information beyond cell counts (25).

Emerging spatial and single-cell biomarkers represent the frontier: spatial transcriptomics and single-cell RNA sequencing resolve the phenotypic heterogeneity of glioma-associated myeloid cells and define reproducible, treatment-modifiable cellular states and ecosystem architectures that are now being translated into candidate response signatures (25, 40). Incorporating these immune and metabolic biomarkers, alongside IDH and MGMT status and immunophenotypic cohort data, into trial design offers a path to enrich for likely responders and to monitor pharmacodynamic effects in real time (9, 12).

## Translational barriers and rationale for combination strategies

Despite a strong biological rationale, several barriers hinder translation: pronounced intratumoral and interpatient heterogeneity layered on IDH/MGMT context; corticosteroid use; restricted access to longitudinal tumor sampling; the privileged but compromised CNS compartment; and reliance on survival endpoints that fail to capture immune reprogramming (9, 10, 41). Future trials should integrate immune and metabolic biomarkers, prioritize rational combinations, align therapeutic timing with standard-of-care interventions (particularly radiotherapy), and consider neoadjuvant windows that permit on-treatment tissue assessment.

## Discussion

The failure of immune checkpoint inhibitors in GBM, now confirmed across CheckMate 143, 498, and 548, reflects not an absence of immunogenicity per se but the dominance of a highly organized, metabolically enforced immunosuppressive microenvironment in a tumor that is intrinsically antigen-poor (3, 37, 38). The microglia–MDSC–Treg axis is a central hub, but it operates within a broader network that includes dysfunctional dendritic and NK cells, exhausted and bone-marrow-sequestered CD8^+^ T cells, immunosuppressive neutrophils, suppressive reactive astrocytes, and an interlocking metabolic barrier built from tryptophan, adenosine, arginine, lactate, and glutamine pathways (8, 25). Strategies that disrupt only one node are unlikely to achieve durable benefit, which is precisely the lesson of single-agent checkpoint and CSF1R inhibition.

These lessons map directly onto the three axes defined at the outset. Durable benefit will require combinations that act simultaneously on the myeloid compartment, the metabolic microenvironment, and the antigenic or inflammatory state of the tumor, because relieving any single axis leaves the others intact; the radiotherapy-anchored CD47/PD-L1/STING nanoparticle illustrates this, if only preclinically (30). Radiotherapy itself should be treated not as a neutral backbone but as an active, double-edged immunomodulator whose dose, fractionation, and timing can be engineered to favor antigen release and STING activation while its compensatory systemic and intratumoral suppressive programs are pharmacologically blocked (30, 42).

Patient selection, in turn, must move beyond histology to incorporate IDH and MGMT status together with immune and metabolic biomarkers measured by multiplex imaging, spatial transcriptomics, and single-cell sequencing, so that each combination reaches the patients whose microenvironment it is designed to modify, ideally within neoadjuvant designs that yield on-treatment tissue (9, 12, 13, 25, 40).

This review has limitations. Its narrative format, while appropriate for an integrative synthesis, does not apply a formal systematic protocol, and the relative scarcity of GBM-specific quantitative data, particularly for MDSC subsets and spatially resolved metabolic measurements, means some inferences are extrapolated from related settings and should be interpreted with caution.

## Conclusion

Glioblastoma remains a formidable therapeutic challenge driven in large part by a deeply immunosuppressive, metabolically enforced, and molecularly heterogeneous tumor microenvironment in which myeloid-driven immune regulation, metabolic reprogramming, and treatment-induced immune remodeling reinforce one another. Targeting the microglia–MDSC–Treg axis within its broader cellular and metabolic network, through integrated, biomarker-guided combinations that exploit rather than ignore radiotherapy and that are stratified by IDH and MGMT status, represents the most promising path beyond current standard of care. Continued progress will require rational combination therapies, biomarker-driven trial design incorporating spatial and single-cell immunoprofiling, and a shift toward immune reprogramming as a measurable therapeutic goal.

The central message of this review can be stated simply. Immune suppression in glioblastoma is not a single defect but a self-reinforcing circuit in which myeloid-driven immune regulation, metabolic reprogramming, and treatment-induced immune remodeling sustain one another, so that relieving any one axis leaves the others intact. Durable benefit is therefore unlikely to come from single-agent immunotherapy and will instead require rational, biomarker-guided combinations that target several interconnected mechanisms of immune suppression at once, with radiotherapy treated as an active component of the regimen rather than a neutral backbone.
